# Inheritance patterns of ATCCT repeat interruptions in spinocerebellar ataxia type 10 (SCA10) expansions

**DOI:** 10.1371/journal.pone.0175958

**Published:** 2017-04-19

**Authors:** Ivette Landrian, Karen N. McFarland, Jilin Liu, Connie J. Mulligan, Astrid Rasmussen, Tetsuo Ashizawa

**Affiliations:** 1 Department of Neurology, College of Medicine, and the McKnight Brain Institute, University of Florida, Gainesville, Florida, United States of America; 2 Center for Translational Research in Neurodegenerative Disease, The University of Florida, Gainesville, Florida, United States of America; 3 Department of Anthropology, College of Liberal Arts and Sciences, University of Florida, Gainesville, Florida, United States of America; 4 Genetics Institute, University of Florida, Gainesville, Florida, United States of America; 5 Arthritis and Clinical Immunology Research Program, Oklahoma Medical Research Foundation, Oklahoma City, Oklahoma, United States of America; University of Valencia, SPAIN

## Abstract

Spinocerebellar ataxia type 10 (SCA10), an autosomal dominant cerebellar ataxia disorder, is caused by a non-coding ATTCT microsatellite repeat expansion in the ataxin 10 gene. In a subset of SCA10 families, the 5’-end of the repeat expansion contains a complex sequence of penta- and heptanucleotide interruption motifs which is followed by a pure tract of tandem ATCCT repeats of unknown length at its 3’-end. Intriguingly, expansions that carry these interruption motifs correlate with an epileptic seizure phenotype and are unstable despite the theory that interruptions are expected to stabilize expanded repeats. To examine the apparent contradiction of unstable, interruption-positive SCA10 expansion alleles and to determine whether the instability originates outside of the interrupted region, we sequenced approximately 1 kb of the 5’-end of SCA10 expansions using the ATCCT-PCR product in individuals across multiple generations from four SCA10 families. We found that the greatest instability within this region occurred in paternal transmissions of the allele in stretches of pure ATTCT motifs while the intervening interrupted sequences were stable. Overall, the ATCCT interruption changes by only one to three repeat units and therefore cannot account for the instability across the length of the disease allele. We conclude that the AT-rich interruptions locally stabilize the SCA10 expansion at the 5’-end but do not completely abolish instability across the entire span of the expansion. In addition, analysis of the interruption alleles across these families support a parsimonious single origin of the mutation with a shared distant ancestor.

## Introduction

The ATTCT pentanucleotide repeat expansion in intron 9 of the ataxin 10 (*ATXN10*) gene on chromosome 22q13.3 causes the neurodegenerative disorder spinocerebellar ataxia type 10 (SCA10) [1]. The ATTCT repeat is normally polymorphic and ranges from 9 to 32 ATTCT repeats in the general population [1, 2] but can expand up to 4,500 repeats in SCA10 patients [1]. SCA10 symptoms include progressive cerebellar ataxia with variable extracerebellar phenotypes—the best characterized of which is epilepsy [3].

In some SCA10 patients, the 5’-end of the repeat expansion contains a series of complex penta- and heptanucleotide repeat interruption motifs bounded at the 3’-end by an ATCCT tandem repeat [4–7]. These interruption sequences are found only in a subset of SCA10 patients and strongly correlate with the presence of epileptic seizures and, surprisingly, with increased intergenerational repeat instability [5, 6]. Generally, interruptions in expansions are thought to act as an insulator or stabilizer against further instability as seen in fragile X pre-mutation expansions [8–10] and in SCA1 [11, 12]and SCA2 normal alleles [13, 14] although interruptions within DM1 and SCA8 repeats are also seen in unstable alleles [15–17]. To investigate the evident contradiction of interruption-positive, highly unstable SCA10 expansions, we investigated the stability of the 5’-end interruption sequence over multiple generations by sequencing the ATCCT-PCR fragment in these families.

## Materials and methods

### SCA10 samples

This study was conducted with approval from the University of Florida's IRB (UF IRB#100–2010) where previously collected and existing de-identified samples and data from all members of the C, M, N and Z families were analyzed. The C, M, and N families were originally recruited in Mexico under the approval of the Ethics Committee of the Instituto Nacional de Neurología y Neurocirugía Manuel Velasco Suárez (FWA00008475) for the characterization of the SCA10 mutation. The Z family was recruited under the approval of the Institutional Review Board at the University of Texas Medical Branch, Galveston, TX (H-4499-8892). All individuals provided written informed consent which allowed for the future use of their de-identified data and materials for additional research in the field of hereditary ataxias and microsatellite repeat expansions.

Twenty-six individuals with ATCCT repeat interruptions were previously identified [5, 6] and are from four unrelated SCA10 families of Mexican ancestry. Genomic DNA was extracted from peripheral blood lymphocytes using standard conditions and used in subsequent reactions.

### ATCCT-PCR and sequence analysis

ATCCT repeat interruptions within SCA10 expansions were PCR amplified (ATCCT-PCR) as previously described using the LP-L forward primer (5’-GGAATTCGGCTTAAATATCCAACTAAAAGACTACTAGAATGG-3’) and L2RT reverse primer (5’-TACGCATCCCAGTTTGAGACGG(AGAAT)_6_−3’) [5]. The approximately 1 kb product was gel purified from a 1% agarose gel using the QIAquick Gel Extraction kit (Qiagen). Following gel purification, the PCR products were directly sequenced (in separate sequencing reactions) using the LP-L forward primer and the LF1h forward primer (5’-GGAATTCATTTTCTATTCTATATTCTATTCTATATTCTATTCTATTTTCT-3’) which anneals to the first interruption region at the 5’-end of the SCA10 expansion. Sequencing reactions were carried out at the University of Florida Interdisciplinary Center for Biotechnology Research (ICBR) Sanger sequencing core facility using an ABI 3130 Genetic Analyzer. Overlapping sequencing results from the LP-L and LF1h primers from the same PCR template were aligned using ClustalW2 from the European Bioinformatics Institute (EBI; http://www.ebi.ac.uk/Tools/msa/clustalw2/) [18] to get full sequence of the PCR product.

## Results

### Sequence of ATCCT-PCR products

**Fig 1:**
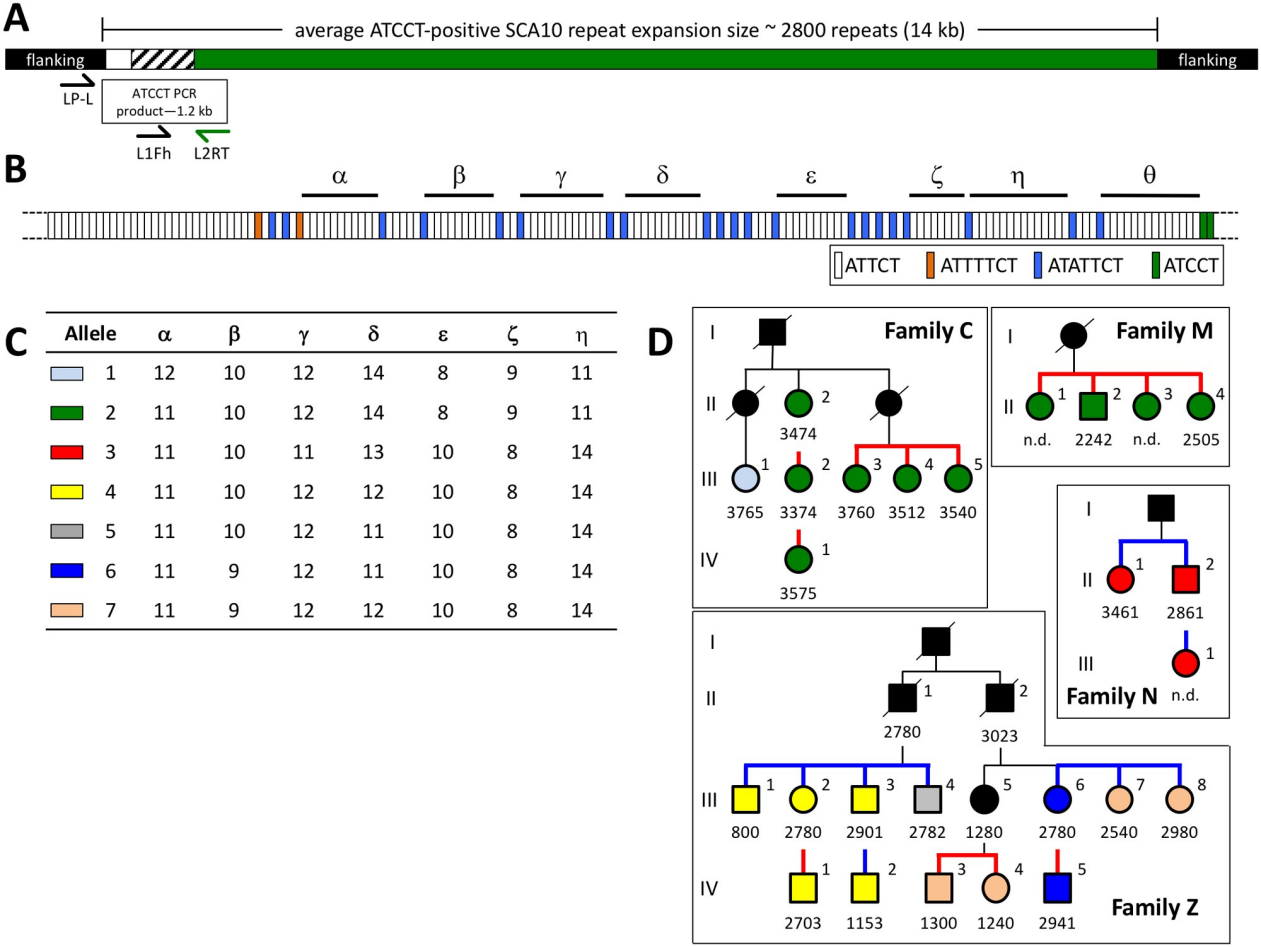
The ATCCT repeat interruption is stable through generations. (A) Schematic of an average length SCA10 expansion allele demonstrating the relative location of the priming sites for the ATCCT PCR amplification and sequencing primers and the relative size of the ATCCT product. White box, ATTCT repeats; hatched black and white box, interruption motifs; green box, presumed pure tract of tandem ATCCT repeat motifs; black box, flanking non-expansion sequences. (B) Detailed schematic of repeat motifs within the ATCCT product. Allele 5 is depicted. White rectangles, ATTCT repeat; orange, ATTTTCT; blue, ATATTCT; green, ATCCT. (C) Seven interruption alleles were observed based on the number of ATTCT repeats observed within each polymorphic stretch (alpha, beta, gamma, delta, epsilon, zeta and eta). (D) SCA10 family pedigrees, only SCA10-positive individuals are shown. Generations are indicated by roman numerals to the left of each pedigree. Square (males) and circles (females) are color-coded by repeat interruption group. Black, undetermined allele; light blue, allele 1; green, allele 2; red, allele 3; yellow, allele 4; grey, allele 5; dark blue, allele 6; tan, allele 7. Numbers below male/female symbols indicate the SCA10 expansion size (in repeat units) determined via Southern blotting; n.d. indicates that the SCA10 expansion size was not determined due to insufficient DNA quality. Thick blue lines, paternal transmissions examined; thick red lines, maternal transmissions examined.

The ATCCT interruption was identified in individuals from four multi-generational SCA10 families [5]. We amplified the ATCCT-PCR product (Fig 1A) containing the repeat interruption motif in 26 individuals and sequenced the approximately 1 kb fragment by Sanger sequencing. In these samples, we found a similar sequence (Fig 1B) as previously described [5].

We found eight variable stretches (indicated by the Greek alphabet alpha, beta, gamma, delta, epsilon, zeta, eta and theta) of pure tandem ATTCT repeats that are polymorphic in our samples, with interspersed invariable regions of heptanucleotide (ATTTTCT and ATATTCT) interruptions (Fig 1B and 1C). The most distal region (region theta) remains incompletely characterized due to an inability to accurately determine the sequence in this region in some of our samples. Likewise, we could not determine the number of ATTCT repeats at the 5’-end of the PCR product due to the short distance between the primer-annealing site and the start of the sequence from the repeat sequence, which was purposefully minimized to improve sequencing distance. The original Sanger sequence trace data is provided as supplemental data files (S1–S40 Files). Examination of the chromatogram of the sequencing traces indicates that there is some heterogeneity in the sequencing reads—particularly at the junction between tandem ATTCT stretches and interrupted regions. Sequence data from sequencing primer L1Fh—which primes at the first interruption region, region alpha) is generally of better quality than that of LP-L which primes on the outside region flanking the start of the repeats. This likely reflects heterogeneity in the first stretch of tandem ATTCT at the far 5’-end of the expansion which is sequenced by the LP-L sequencing primer. All individuals were sequenced with the LP-L primer; however, sequencing the PCR product using the L1Fh primer was not achieved in all samples. Overall heterogeneity in the sequencing reads is unsurprising given that we are directly sequencing PCR products amplified directly from genomic DNA.

Focusing on the first seven polymorphic ATTCT regions, the results from the sequencing data resolved the samples into 7 alleles, denoted 1 through 7 in Fig 1B, see S1 Table. We find that some interruption alleles were unique to a single individual (family C, allele 1; family Z, allele 5); one allele was shared by multiple individuals within a single family (allele 3 in family N) while another allele was shared by multiple individuals in two different families (allele 2 in families C & M). Finally, the largest family (Family Z) was also the most diverse, containing four interruption alleles amongst twelve family members.

### Intergenerational variability of repeat interruptions

We compared the interruption alleles in six parent-child pairs—two paternal (Fig 1D; Family N: II-2 to III-1 and Family Z: III-3 to IV-2) and four maternal transmissions (Fig 1D; Family C: II-2 to III-2; Family C: III-2 to IV-1; Family Z: III-2 to IV-1; Family Z: III-6 to IV-5). While the ATTCT-positive repeat expansions were unstable across the entirety of the expansion length during intergenerational transmission as evidenced by Southern blot analysis (see Fig 1D), the sequence within the interruption region at the 5’-end in our analysis did not change in either paternal or maternal transmissions (see S1 Table). Indeed, this stable germline transmission of the interruption allele was observed across three generations in family C.

Since the number of direct parent-child transmissions was small, we inferred germline stability of the interruption by examining sibling groups who shared a SCA10-positive parent. In our pedigrees, we found three sibling branches where the SCA10 expansion was paternally inherited (Family N, II:1–2 and Family Z, III:1–4 and III:5–8). The shared interruption allele in the siblings of one of these branches (Family N, II:1–2, allele 3) indicated a likely stable inheritance of the interruption allele. Whereas in two other branches we noticed that the siblings did not share the repeat interruption allele—two interruption groups were observed amongst four siblings in one branch (family Z: III-1 through III-4, alleles 4 and 5) and two interruption groups across three siblings in the other branch (family Z: III-6 through III-8, alleles 6 and 7)—indicating unstable transmission of the interruption in at least two instances. In maternal transmissions, there were three sibling branches (Family C, III:3–5, allele 2; Family M, II:1–4, allele 2; and Family Z, IV:3–4, allele 8) and siblings within each branch shared the same interruption allele.

Altogether, we observed two cases of instability in the variable ATTCT repeat allele out of a total of eleven paternal transmissions while we did not observe instability of the interruption allele in any of the thirteen maternal transmission. While this result is not a statistically significant difference between paternal and maternal germline transmissions (Fisher’s exact test, two-sided P = 0.1993), the trend towards SCA10 repeat expansions being more unstable in paternal transmissions is consistent with earlier observations [5].

### Shared origin of SCA10 repeat interruption

**Fig 2:**
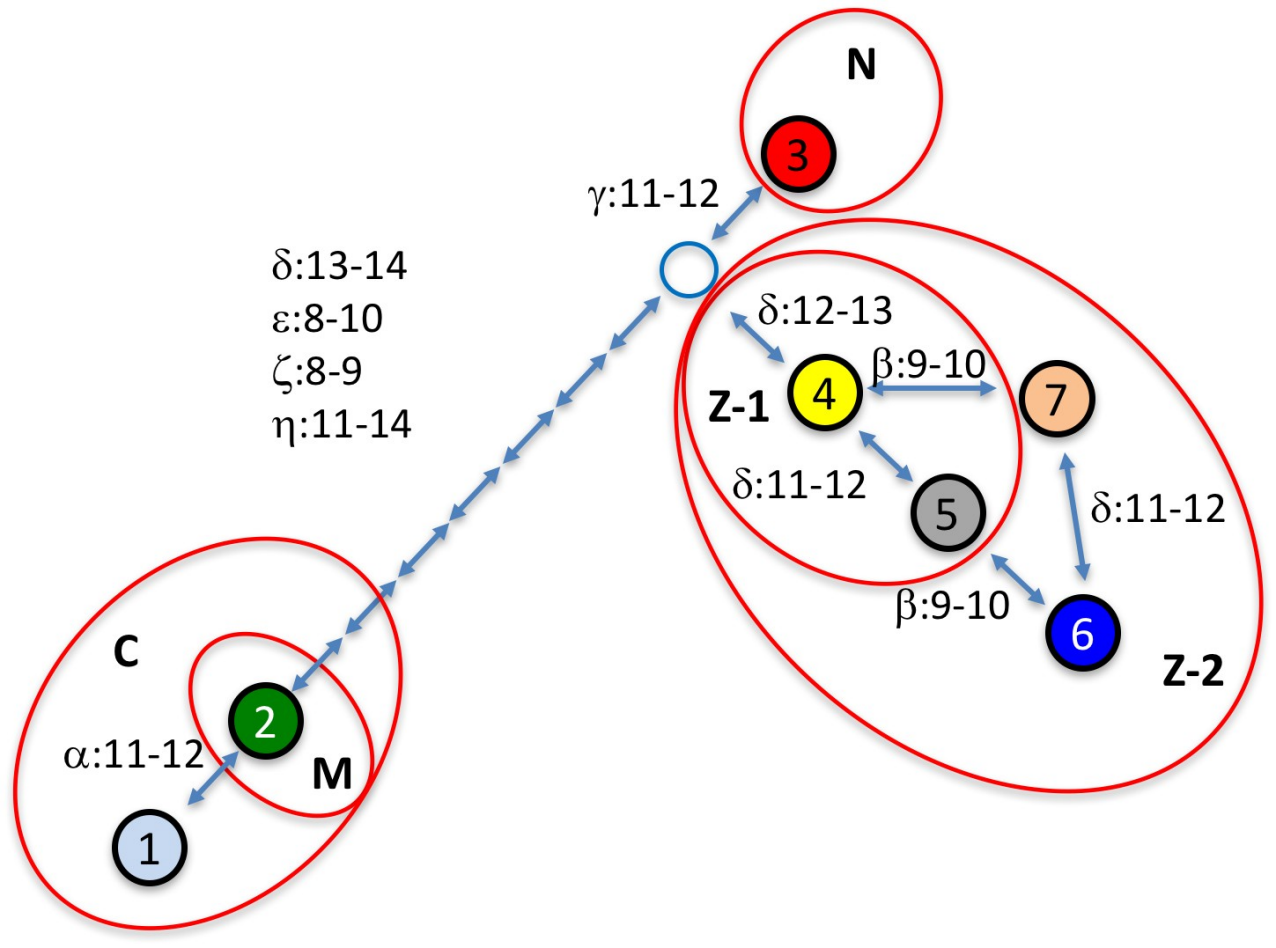
A minimum spanning network depicting the hypothesized evolution of the ATCCT repeat interruption alleles. Filled colored circles and numbers correspond to interruption alleles as in Fig 1. The open blue circle represents a hypothetical allele suggested to exist based on the network. Each bidirectional arrow represents a single repeat unit change between alleles and each arrow notes specific changes. When multiple repeat changes exist between interruption alleles, the order of the repeat changes is not known, i.e. the order of changes between alleles 2 and 3 is not known. The alleles that appear in each family are contained within a red oval and the family (C, M, N, Z) is noted within. The network does not reflect the variation at the distal variable region, theta.

We constructed a minimum spanning network (MSN) of the interruption alleles to illustrate a proposed evolution of these repeat expansion between these families (Fig 2). MSNs are used to visualize relationships among individuals in a dataset and are based on a parsimony principle of minimizing the distance between all individuals to yield the most likely network. The number of times each mutational event occurs is also minimized. Thus, the analysis suggests that all interruption alleles detected in this study share a single, common origin. Furthermore, families C and M form a more closely related group as do families N and Z (i.e. all family members are connected by single repeat mutational steps) while these two groups of families (C and M versus N and Z) are more distantly related (all members from the two groups of families are separated by at least eight single repeat mutational steps). We can do a rough calculation of the minimum time to the most recent common ancestor of the four families based on our observation of four mutations in 31 transmissions (1 mutation/7.75 generations), the presence of at least eight mutations between the two groups of families (common ancestor 31 generations ago) and a generation time of 25 years to yield an estimate of ~775 years.

## Discussion

SCA10 expansions can be highly unstable even though they contain interruption sequences. Typically, interruptions are thought to stabilize repeat stability and are often found in normal alleles of many other repeat expansion loci. Indeed, normal *ATXN10* alleles contain interruption sequences (ATTGT and TTTCT motifs) that are located at the 3-end of the majority of normal alleles [4]. To investigate the contradictory appearance of interruption sequences within unstable SCA10 alleles, we sequenced and characterized the ATCCT-PCR product which amplifies the interruption allele at the 5’-end of the SCA10 expansion.

### Variability in 5’-end interrupted region does not account for overall expansion changes

The presence and sequence of the interrupting motifs was constant across these four families; however, the intervening stretches of tandem ATTCT sequence were variable. Amongst the 26 samples, we determined that there were seven shared interruption alleles based on the polymorphic ATTCT stretches. The interruption alleles were stably inherited in the thirteen maternal transmissions but were unstable in two of the eleven paternal transmissions. In these instances of instability, the difference was slight and usually a matter of a single unit difference in one of the variable ATTCT stretches—an intriguing observation given that these ATTCT repeat tracts are within the range of stable normal alleles.

The cases where the interruption allele was stable were also informative. For instance, the direct grandmother-mother-child lineage in Family C highlights that the 5’-end interruption sequences remain locally stable while the entirety of the expansion allele is unstable (-100 repeat units in the grandmother-to-mother and +201 repeat units in the mother-to-child transmissions). Thus, such large-scale changes in the expansion size of the SCA10 allele must be occurring at the 3’-end of the expansion and outside of the interruption region—a region that we have previously shown to be primarily composed of ATCCC and ATCCT motifs [5, 7].

We can only speculate about the mechanisms of the instability in the pure, variable stretches of ATTCT repeats versus the stabilization provided by ATTTTCT and ATATTCT interruptions. Unpaired structures and various sister strand exchange events have been postulated to explain the instability of the pure ATTCT repeat [19, 20]. However, these mechanisms are likely to introduce a greater perturbation of the overall repeat structure, and the remarkable consistency in the location and sequence of these interruption motifs argues against such a mechanism for instability. Instead, a repeat slippage model where the repeat region forms a slipped-strand structure (S-DNA), as seen for CAG and CGG repeats (SCA1 and FRAXA, respectively [21, 22]) may be a simpler explanation. The ATTCT repeat stretch easily unwinds [23] and the ATTCT strand of the repeat preferentially forms a folded structure [24] which could promote slippage. The interrupting sequences may act as an insulator against instability by having an anchoring effect that prevents slipped strand formation of the ATTCT repeat [25]. Stabilization of the repeat expansion by interrupting sequences has been observed in microsatellite repeats of other repeat expansion disorders [9, 26, 27] which possibly involve repair pathways [28, 29].

Our data highlight the dichotomous nature of interruption-containing SCA10 expansions. The interruption motifs act to locally stabilize the 5’-end of the repeat expansion where relatively small, single repeat unit changes are seen between generations. However, these interruptions exert little effect on the overall instability of SCA10 expansions where intergenerational changes in allele size can often exceed hundreds of repeat units. In addition, analysis of the interrupted SCA10 expansion alleles via a minimal spanning network found that these families share a common, distant origin and lend further support to the idea of a shared origin for all SCA10 expansion mutations [30]. Changes in the interruption alleles described here will help delineate the origin and evolution of the SCA10 expansion.

## Supporting information

S1 Table Interruption alleles of SCA10 expansions. For each individual, the number of ATTCT repeats in each of the variable regions (alpha through eta) are given. Region theta is not included as this region cannot be completely characterized. The nomenclature of each individual corresponds with the pedigrees in Fig 1D. Expansions sizes are given in the number of repeat units. In some cases, the expansion size could not be determined by Southern blot (indicated by n.a.). Change in the SCA10 expansion size during in germline transmission was calculated by subtracting the expansion size of the child from that of the parent. In some instances, this information is not available (n.a.) if the expansion size of the child or the parent (or both) is not known.
(DOCX)

S1 File Sanger sequencing trace data of the ATCCT-PCR product from C-II-2 using sequencing primer LP-L. (SCF)

S2 File Sanger sequencing trace data of the ATCCT-PCR product from C-II-2 using sequencing primer L1Fh. (SCF)

S3 File Sanger sequencing trace data of the ATCCT-PCR product from C-III-1 using sequencing primer LP-L. (SCF)

S4 File Sanger sequencing trace data of the ATCCT-PCR product from C-III-1 using sequencing primer L1Fh. (SCF)

S5 File Sanger sequencing trace data of the ATCCT-PCR product from C-III-2 using sequencing primer LP-L. (SCF)

S6 File Sanger sequencing trace data of the ATCCT-PCR product from C-III-2 using sequencing primer L1Fh. (SCF)

S7 File Sanger sequencing trace data of the ATCCT-PCR product from C-III-3 using sequencing primer LP-L. (SCF)

S8 File Sanger sequencing trace data of the ATCCT-PCR product from C-III-3 using sequencing primer L1Fh. (SCF)

S9 File Sanger sequencing trace data of the ATCCT-PCR product from C-III-4 using sequencing primer LP-L. (SCF)

S10 File Sanger sequencing trace data of the ATCCT-PCR product from C-III-4 using sequencing primer L1Fh. (SCF)

S11 File Sanger sequencing trace data of the ATCCT-PCR product from C-III-5 using sequencing primer LP-L. (SCF)

S12 File Sanger sequencing trace data of the ATCCT-PCR product from C-III-5 using sequencing primer L1Fh. (SCF)

S13 File Sanger sequencing trace data of the ATCCT-PCR product from C-IV-1 using sequencing primer LP-L. (SCF)

S14 File Sanger sequencing trace data of the ATCCT-PCR product from M-II-1 using sequencing primer LP-L. (SCF)

S15 File Sanger sequencing trace data of the ATCCT-PCR product from M-II-1 using sequencing primer L1Fh. (SCF)

S16 File Sanger sequencing trace data of the ATCCT-PCR product from M-II-2 using sequencing primer LP-L. (SCF)

S17 File Sanger sequencing trace data of the ATCCT-PCR product from M-II-3 using sequencing primer LP-L. (SCF)

S18 File Sanger sequencing trace data of the ATCCT-PCR product from M-II-3 using sequencing primer L1Fh. (SCF)

S19 File Sanger sequencing trace data of the ATCCT-PCR product from M-II-4 using sequencing primer LP-L. (SCF)

S20 File Sanger sequencing trace data of the ATCCT-PCR product from N-II-1 using sequencing primer LP-L. (SCF)

S21 File Sanger sequencing trace data of the ATCCT-PCR product from N-II-1 using sequencing primer L1Fh. (SCF)

S22 File Sanger sequencing trace data of the ATCCT-PCR product from N-II-2 using sequencing primer LP-L. (SCF)

S23 File Sanger sequencing trace data of the ATCCT-PCR product from N-II-2 using sequencing primer L1Fh. (SCF)

S24 File Sanger sequencing trace data of the ATCCT-PCR product from N-III-1 using sequencing primer LP-L. (SCF)

S25 File Sanger sequencing trace data of the ATCCT-PCR product from Z-III-1 using sequencing primer LP-L. (SCF)

S26 File Sanger sequencing trace data of the ATCCT-PCR product from Z-III-1 using sequencing primer L1Fh. (SCF)

S27 File Sanger sequencing trace data of the ATCCT-PCR product from Z-III-2 using sequencing primer LP-L. (SCF)

S28 File Sanger sequencing trace data of the ATCCT-PCR product from Z-III-3 using sequencing primer LP-L. (SCF)

S29 File Sanger sequencing trace data of the ATCCT-PCR product from Z-III-4 using sequencing primer LP-L. (SCF)

S30 File Sanger sequencing trace data of the ATCCT-PCR product from Z-III-4 using sequencing primer L1Fh. (SCF)

S31 File Sanger sequencing trace data of the ATCCT-PCR product from Z-III-6 using sequencing primer LP-L. (SCF)

S32 File Sanger sequencing trace data of the ATCCT-PCR product from Z-III-7 using sequencing primer LP-L. (SCF)

S33 File Sanger sequencing trace data of the ATCCT-PCR product from Z-III-8 using sequencing primer LP-L. (SCF)

S34 File Sanger sequencing trace data of the ATCCT-PCR product from Z-IV-1 using sequencing primer LP-L. (SCF)

S35 File Sanger sequencing trace data of the ATCCT-PCR product from Z-IV-1 using sequencing primer L1Fh. (SCF)

S36 File Sanger sequencing trace data of the ATCCT-PCR product from Z-IV-2 using sequencing primer LP-L. (SCF)

S37 File Sanger sequencing trace data of the ATCCT-PCR product from Z-IV-2 using sequencing primer L1Fh. (SCF)

S38 File Sanger sequencing trace data of the ATCCT-PCR product from Z-IV-3 using sequencing primer LP-L. (SCF)

S39 File Sanger sequencing trace data of the ATCCT-PCR product from Z-IV-4 using sequencing primer LP-L. (SCF)

S40 File Sanger sequencing trace data of the ATCCT-PCR product from Z-IV-5 using sequencing primer LP-L. (SCF)
