# Supplementary material for: Inheritance patterns of ATCCT repeat interruptions in spinocerebellar ataxia type 10 (SCA10) expansions
Source: PLoS One. 2017 Apr 19;12(4):e0175958. doi: 10.1371/journal.pone.0175958 (PMC5397023; doi:10.1371/journal.pone.0175958)
Supplement: S1 Table — For each individual, the number of ATTCT repeats in each of the variable regions (alpha through eta) are given. Region theta is not included as this region cannot be completely characterized. The nomenclature of each individual corresponds with the pedigrees in Fig 1D. Expansions sizes are given in the number of repeat units. In some cases, the expansion size could not be determined by Southern blot (indicated by n.a.). Change in the SCA10 expansion size during in germline transmission was calculated by subtracting the expansion size of the child from that of the parent. In some instances, this information is not available (n.a.) if the expansion size of the child or the parent (or both) is not known. (DOCX) [file pone.0175958.s001.docx]

# S1 Table

## Interruption alleles of SCA10 expansions

| **Family** | **Individual** | **Expansion Size** | **Change in Overall Expansion Size** | **Change in interrupted region** | **Inheritance** | **Allele** | **Number of ATTCT repeats in variable region** | | | | | | |
| --- | --- | --- | --- | --- | --- | --- | --- | --- | --- | --- | --- | --- | --- |
|  |  |  |  |  |  |  | **α** | **β** | **γ** | **δ** | **ε** | **ζ** | **η** |
| C | III-1 | 3765 | n.a. | n.a. | M | 1 | 12 | 10 | 12 | 14 | 8 | 9 | 11 |
| C | II-2 | 3474 | n.a. | n.a. | M | 2 | 11 | 10 | 12 | 14 | 8 | 9 | 11 |
| C | III-2 | 3374 | -100 | 0 | M | 2 | 11 | 10 | 12 | 14 | 8 | 9 | 11 |
| C | III-3 | 3760 | n.a. | n.a. | M | 2 | 11 | 10 | 12 | 14 | 8 | 9 | 11 |
| C | III-4 | 3512 | n.a. | n.a. | M | 2 | 11 | 10 | 12 | 14 | 8 | 9 | 11 |
| C | III-5 | 3540 | n.a. | n.a. | M | 2 | 11 | 10 | 12 | 14 | 8 | 9 | 11 |
| C | IV-1 | 3575 | +201 | 0 | M | 2 | 11 | 10 | 12 | 14 | 8 | 9 | 11 |
|  |  |  |  |  |  |  |  |  |  |  |  |  |  |
| M | II-1 | n.a. | n.a. | n.a. | M | 2 | 11 | 10 | 12 | 14 | 8 | 9 | 11 |
| M | II-2 | 2242 | n.a. | n.a. | M | 2 | 11 | 10 | 12 | 14 | 8 | 9 | 11 |
| M | II-3 | n.a. | n.a. | n.a. | M | 2 | 11 | 10 | 12 | 14 | 8 | 9 | 11 |
| M | II-4 | 2505 | n.a. | n.a. | M | 2 | 11 | 10 | 12 | 14 | 8 | 9 | 11 |
|  |  |  |  |  |  |  |  |  |  |  |  |  |  |
| N | II-1 | 3461 | n.a. | n.a. | P | 3 | 11 | 10 | 11 | 13 | 10 | 8 | 14 |
| N | II-2 | 2861 | n.a. | n.a. | P | 3 | 11 | 10 | 11 | 13 | 10 | 8 | 14 |
| N | III-1 | n.a. | n.a. | 0 | P | 3 | 11 | 10 | 11 | 13 | 10 | 8 | 14 |
|  |  |  |  |  |  |  |  |  |  |  |  |  |  |
| Z | III-1 | 800 | -1980 | n.a. | P | 4 | 11 | 10 | 12 | 12 | 10 | 8 | 14 |
| Z | III-2 | 2780 | 0 | n.a. | P | 4 | 11 | 10 | 12 | 12 | 10 | 8 | 14 |
| Z | III-3 | 2901 | +121 | n.a. | P | 4 | 11 | 10 | 12 | 12 | 10 | 8 | 14 |
| Z | IV-1 | 2703 | -77 | 0 | M | 4 | 11 | 10 | 12 | 12 | 10 | 8 | 14 |
| Z | IV-2 | 1153 | -1748 | 0 | P | 4 | 11 | 10 | 12 | 12 | 10 | 8 | 14 |
| Z | III-4 | 2782 | +2 | n.a. | M | 5 | 11 | 10 | 12 | 11 | 10 | 8 | 14 |
| Z | III-6 | 2780 | -243 | n.a. | P | 6 | 11 | 9 | 12 | 11 | 10 | 8 | 14 |
| Z | IV-5 | 2941 | +161 | 0 | M | 6 | 11 | 9 | 12 | 11 | 10 | 8 | 14 |
| Z | III-7 | 2540 | -483 | n.a. | P | 7 | 11 | 9 | 12 | 12 | 10 | 8 | 14 |
| Z | III-8 | 2980 | -43 | n.a. | P | 7 | 11 | 9 | 12 | 12 | 10 | 8 | 14 |
| Z | IV-3 | 1300 | +20 | n.a. | M | 7 | 11 | 9 | 12 | 12 | 10 | 8 | 14 |
| Z | IV-4 | 1240 | -40 | n.a. | M | 7 | 11 | 9 | 12 | 12 | 10 | 8 | 14 |
